# Mr. Robert William Coe

**Published:** 1910-08-20

**Authors:** 


### Obituary.

The death is announced of Mr. Robert William Coe, who died at Bristol, aged eighty-nine. Mr. Coe, who qualified from St. George's in 1844, in 1852 took the English Fellowship, and was therefore the second oldest Fellow of the Royal College of Surgeons. He was a consulting surgeon to the Bristol General Hospital, and to the Hospital for Children, Surgeon to the Lock Hospital, and late Lecturer on Surgery to the Bristol Medical School, and had contributed largely to the professional press.

